# Secondary Education in COVID Lockdown: More Anxious and Less Creative—Maybe Not?

**DOI:** 10.3389/fpsyg.2021.613055

**Published:** 2021-02-22

**Authors:** Timothy J. Patston, JohnPaul Kennedy, Wayne Jaeschke, Hansika Kapoor, Simon N. Leonard, David H. Cropley, James C. Kaufman

**Affiliations:** ^1^UniSA: STEM, University of South Australia, Adelaide, SA, Australia; ^2^Centre for Creative Education, Geelong Grammar School, Geelong, VIC, Australia; ^3^UniSA: Education Futures, University of South Australia, Adelaide, SA, Australia; ^4^Centre for Change and Complexity in Learning (C^*3*^L), University of South Australia, Adelaide, SA, Australia; ^5^Trinity Research Institute, Trinity College, Adelaide, SA, Australia; ^6^Neag School of Education, University of Connecticut, Storrs, CT, United States; ^7^Monk Prayogshala, Mumbai, India

**Keywords:** COVID-19 pandemic, creativity, resiliency, school attitudes survey, secondary education, student attitudinal profiles, attitude surveying

## Abstract

Secondary education around the world has been significantly disrupted by covid-19. Students have been forced into new ways of independent learning, often using remote technologies, but without the social nuances and direct teacher interactions of a normal classroom environment. Using data from the School Attitudes Survey—which surveys students regarding the perceived level of difficulty, anxiety level, self-efficacy, enjoyability, subject relevance, and opportunities for creativity with regards to each of their school subjects—this study examines students' responses to this disruption from two very different schools with two very different experiences of the pandemic. This paper reports on the composite attitudinal profiles of students in the senior secondary levels at each school (Years 10–12, *n* = 834). The findings challenged our expectation that the increased difficulty and anxiety caused by the disruption would reduce perceived opportunities for creativity. Indeed, our analyses showed that the students at both schools demonstrated generally positive attitudes toward their learning and strongly associated opportunities for creativity with other attitudinal constructs including enjoyability, subject relevance, and self-efficacy. These complex associations made by the students appear to have buffered the impacts of the disruption, and they may even have supported creative resilience.

## Introduction

The importance of developing and building complex competencies through education has become a staple of global education policy development. The OECD, for instance, has highlighted the significance of creativity in its work on the “transformative competencies” needed from education in the twenty-first century (OECD, 2019). The best ways to develop competencies such as creativity within formal education, however, remain a matter of intense research interest (Vincent-Lancrin et al., 2019) and debate (Cropley and Patston, 2019). It is not surprising, then, that little is known about the interaction of creativity in schooling and a crisis such as covid-19.

Historical evidence suggests that periods of crisis have been crucibles for creativity—World War Two (Chin, 2019) and the Sputnik Shock in the US after 1957 (Steeves et al., 2009), for instance, are often credited with stimulating many novel advances in computing, aerospace, and other areas. It is not clear, however, if the link between a larger crisis and creative growth can be carried over into education for creativity. Indeed, although there is ample evidence that creativity thrives under constraints (Acar et al., 2019), there is sparse evidence as to if and how creativity operates under constraints in children (Kupers et al., 2019). There is no doubt that the covid-19 pandemic has imposed constraints on learning and teaching. However, has this been helpful or a hindrance to adolescent creativity in online learning? The covid-19 crisis has in some ways narrowed the pedagogical options available to teachers with a global trend toward exclusive online learning, often with little time for preparation. Hence, the increased difficulties around study may have suppressed at least the student-perceived opportunities for creativity. The cases we will consider in this paper are focused on senior secondary students toward the end of their schooling. These students are preparing for the formal high-stakes examinations that are held at the end of Year 12 which are, in Australia, a major determinant of university matriculation.

Outside of the context of schooling, evidence on the impact of more generalized stress or anxiety on children's creativity is sparse (Wu and Chiou, 2008). However, there is some evidence that creativity can increase resiliency (Metzl, 2009), relieve burdens (Goncalo et al., 2015), reduce personal stress (Byron et al., 2010), and generally enhance meaning and positive mental health outcomes (Kaufman, 2018), even at a tumultuous time such as this (Fegert et al., 2020). There is increasing evidence of a strong link between creativity and self-efficacy, from Bandura's (1982) assertion that self-efficacy is an important cognitive mediator of action, followed by Bandura (1997), evolving their thinking and suggesting a causal link to creative performance. Tierney and Farmer (2002), created a scale to assess creative self-efficacy. Beghetto (2006) refined this and used it in an education setting. This current investigation adds to this evidence base by exploring student perceptions, their affective response to the pandemic, including their perception of their opportunities to respond creatively within their schooling. Is student creativity under covid-19 in keeping with meta-analytic findings (Byron et al., 2010) of a curvilinear relationship between evaluative stress and creativity, such that low evaluative contexts increased creative performance (compared to control conditions), whereas highly evaluative contexts decreased creative performance? Or did students respond creatively to the constraints of online learning?

This exploratory study investigated four key areas. Firstly, students' self-rating of creativity in their independent learning under covid-19. Secondly, how these attitudes vary between schools that have experienced very different impacts of the pandemic. Thirdly, we investigated other attitudinal constructs with the purpose of identifying areas of commonality across key learning areas of the curriculum. Finally, we aimed to investigate the domain specificities of students' perception of creativity between key learning areas. This paper contributes to the field of creativity research by showing that students' concepts of creativity are not domain general but rather demonstrate a degree of domain specificity.

## COVID-19 and Two Australian Schools

The covid-19 pandemic has had a significant impact upon society and education globally. Aside from the wider public health and economic impacts, it has seen many educational providers around the world forced to rapidly pivot to online learning during 2020 (Basilaia and Kvavadze, 2020; OECD, 2020). Although higher education already operates extensively in the online space, the transition to online-mediated distance learning in schooling has been as extraordinary as it has been unexpected, with Hong Kong during the SARS outbreak of 2003 offering perhaps the only significant precedent (Fox, 2004).

This paper explores the experience of the early days of the covid-19 pandemic as self-reported by the students of two schools in Australia. It does so using data from the Schools Attitude Survey (SAS, Kennedy et al., 2016), a validated survey instrument that collects self-report data on student attitudes toward perceived anxiety, opportunity for creativity, difficulty, enjoyability, intentions, subject relevance, self-efficacy, career usefulness, and personal usefulness with regards to each of their subjects at a point in time. The data used are a snapshot taken in the midst of an unprecedented crisis. These students in Year 10–12 (generally 15–18 years old) were attending schools in which, prior to the covid-19 crisis, online learning was present, but did not form a significant part of the learning design at either school; the predominant modality for learning and teaching in both schools was face-to-face.

At the start of the academic year in January, these students were expecting a school year dominated by traditional face-to-face learning, and most would have been looking ahead to prospects within a relatively strong labor market or the vibrant Australian university system. Alternatively, they may have been thinking of the now common “gap year” between school and university with a year spent traveling and possibly working abroad. By March these students were living a very different reality. Australia's international borders, and even many internal state borders, were closing to all but essential travel. Thoughts of travel and study overseas or even interstate were, at best, uncertain. With unemployment already tipping 13% and youth unemployment climbing much higher, thoughts of working at home were no more definite. To add to this picture the Australian university sector, heavily reliant on international students, was in chaos having shed thousands of academic jobs. On seemingly every front, the “certainties” of their future had been upended to a degree perhaps only surpassed by the World Wars.

The coeducational schools we have included in this study are very different institutions, both before and during the pandemic. One, which we will call Green Tree Frog School (GTFS), is predominantly a non-academically selective boarding school with many students of high socioeconomic status and upwards of 90% of students proceed into higher education. In contrast, the school we will call Corroboree Frog College (CFC) is a non-academically selective day student only school that attracts students from a wide socioeconomic background. Around two-thirds of the households with students at the school sit within the middle two quartiles of Australian household income, but students from the top and bottom quartiles also attend CFC. Typically, around 60–70% of CFC students' progress to university.

The mid-pandemic experience for these two schools has also been quite different. GTFS is located in the Australian state with the largest outbreak of the virus. In late March, toward the end of the first term of the school year, it initially closed to face-to-face teaching for 9 weeks. Upon returning to campus its boarding houses necessitated the implementation of quite extreme distancing and hygiene protocols and a further outbreak of the virus ended face-to-face teaching once again as we write this paper. CFC, on the other hand, is located in a state where the virus was virtually eliminated and which has experienced no further significant outbreaks. This has meant that school life was able to return to something very close to normal after an extra week of school holidays in April and only 1 week of online-mediated distance learning. CFC students have still had to deal with the social, emotional, and economic crisis that is surrounding them, but the impacts on their day-to-day activities have been limited. It is reasonable to suggest therefore that GTFS has experienced the full impact of the pandemic while the experience at CTC has been much less pronounced.

More greatly impacted, GTFS took a uniform approach to online learning. Within that structure students were encouraged by teachers to develop individual methods of information transfer, information consolidation, and information retrieval. Mindful that all the external final-year exams conducted by the state and international authorities were still being conducted in handwritten form, students were also encouraged to maintain their handwriting skills and upload photos of their physical work to the digital platform. This standardized format had been in place for 5 weeks when the survey reported in the paper was conducted.

CFC adopted a more flexible approach that encouraged teachers to individually adopt various technologies and to take the opportunity to explore novel approaches to online learning. The intent and nature of online learning varied across year levels and different parts of the multi-campus school, according to the needs of students. Students typically engaged in a weekly web-conference for each subject to support online learning activities and to provide opportunities for collaboration and connection to peers and teachers.

## Creativity

It is easy to assume that there is little consensus on the definition of creativity, yet there is actually widespread agreement on core concepts (Cropley, 2015). From early investigations (see, for eample, Guilford, 1950; Barron, 1955) to modern conceptions (see Diedrich et al., 2015; Kaufman, 2016), definitions of creativity have had two essential components. Creativity involves originality or novelty, and it must also involve task appropriateness (or usefulness). In this formulation, something is creative if it is new *and* it is fit for its desired purpose (Simonton, 2012).

It has long been established that creativity can be a teachable and learnable skill within schools (for a meta-analysis see Scott et al., 2004). It is for this reason that it is being included in many global curricula as an essential twenty-first century skill (Kupers et al., 2019).

Our interest in this paper, though, is on student perceptions. As we will detail below, with respect to creativity, students were asked simply to rate the extent to which they were able to “develop new and useful ways of independent learning” in each of their school subjects. That is, the response is very much about the opportunities to engage in creativity now, as a part of independent online learning as school students, and not about learning to be more creative in the future. However, unlike the other attitudes measured in the survey, creativity is teachable and learnable (Beghetto and Kaufman, 2010). If the students in this study are already developing new and useful ways of independent learning, then teachers can further build this competency over time. Attitudes such as enjoyability or anxiety are able to be discussed in education but cannot be explicitly taught.

## Methods

### The School Attitudes Survey

**Figure 1:**
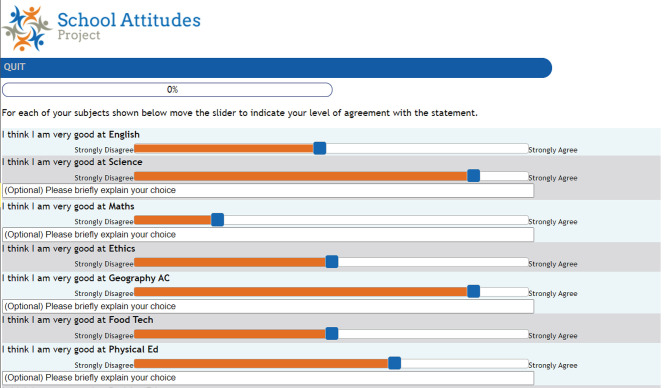
Screenshot of the school attitudes survey interface.

Attitudes are multidimensional constructs. Within the context of schooling it is reasonable to suggest that the distinct aspects of a student's attitudes toward their school subjects may develop at different rates and in response to different stimuli for the student's different subjects. The School Attitude Survey (SAS) was developed from the School Science Attitude Survey (Kennedy et al., 2016) and measures this nuanced approach to a student's attitudes or perceptions toward their subjects against nine Attitudinal Factors: Subject Anxiety, Creativity, Perceived Difficulty, Enjoyability, Intentions, Subject Relevance, Self-Efficacy, Career Usefulness, and Personal Usefulness. The SAS uses a digital interface to present participants with a slider input for each of their school subjects (Figure 1). These slider inputs form a visual analog scale from −50 to +50 and each of the general statements is presented on a separate screen (see Supplementary Material for the item wording). The Attitudinal Factors Anxiety and Difficulty are both reverse keyed in the analyses such that positive values represent a more desirable outcome, i.e., a positive rating for anxiety represents a low-level of anxiety (relaxed students are the desired outcome) and a positive rating for difficulty represents a low-level of difficulty (students who are not struggling is the desired outcome).

A student's mean attitude rating for each attitudinal factor across all of their subjects is calculated and is known as the student's Composite Attitude Rating (CAR). A student's CAR can be thought of as a measure of their average attitude to the academic aspects of school as a whole. Subject Attitude Ratings (SAR) can then be calculated by subtracting a student's CAR for a specific attitudinal factor from their raw attitude rating for the individual subject for that same attitudinal factor. A SAR could therefore theoretically fall in the range −100 to +100. A student's SAR can be thought of as a measure of their attitudes toward a single subject area in comparison to their attitude toward school as a whole. Table 1 shows the calculation and relationship between raw, composite, and subject attitude ratings for a hypothetical student.

**Table 1:** Hypothetical conversion of participant's raw attitude ratings into composite and subject attitude ratings for a partial attitudinal profile.

**Attitudinal factor**	**Subject raw rating**			**Composite attitude rating**	**Subject attitude rating**		
	**English**	**Science**	**Mathematics**		**English**	**Science**	**Mathematics**
Subject Anxiety^*^	+20	+10	+15	+15.0	+5.0	−5.0	0.0
Creativity	+30	−10	+20	+13.3	+16.7	−23.3	+6.7
Difficulty^*^	−20	+10	−10	−6.7	−13.3	+16.7	−3.3

* *Indicates that this scale is reverse keyed*.

All Year 10, 11 and 12 students (15–18 year-olds) at both schools were invited to contribute data to the SAS instrument via a personalized email link in the penultimate week of Term 2 2020 (late June 2020). Students were provided time during a nominated class to complete the SAS instrument using their own computer or other device (e.g., iPad). Ethics approval was granted by the University of South Australia Human Research Ethics Committee and the participants' legal guardians provided informed opt-in consent for the study.

The profile of the two school samples is shown in Table 2. These profiles represent a response rate of 67% (GTFS) and 51% (CFC) for the two schools. GTFS has been explicitly incorporating the ideas and principles of creative education into its curriculum for around 3 years. CFC has recently shifted its strategic intent to improve student attainment of twenty-first century capabilities and has started to invest in resources and professional development for teachers to foster students' creativity.

**Table 2:** Student sample profile for Corroboree Frog College and Green Tree Frog School.

**Student gender**	**Corroboree frog college**				**Green tree frog school**				**Grand total**
	**Year 10**	**Year 11**	**Year 12**	**Total**	**Year 10**	**Year 11**	**Year 12**	**Total**	
Male	13	75	80	**168**	53	55	76	**184**	**352**
Female	29	101	89	**219**	82	75	106	**263**	**482**
Total	42	176	169	**387**	135	130	182	**447**	**834**
Grand Total	**177**	**306**	**351**	**834**	**177**	**306**	**351**	**834**	

Patterns of interest, both within and between schools, were identified and investigated further through the use of boxplot comparisons, ANOVA and correlation analysis using various packages available in the R statistical environment (R Core Team, 2018). Quantile-Quantile plots and the Shapiro-Wilk W statistic were utilized to ensure sufficient normality in the data for the use of parametric analysis. Pearson's product moment coefficient *(r)* was used to analyze the strength of correlations between attitudinal factors in an attitude profile. Tukey's Honest Significance Test, with a 95% confidence interval, was used to determine the extent of the differences in the population means identified by the ANOVA results and eta-squared *(*η^2^*)* was used as a measure of effect size. The relative strength of the different relationships will be interpreted using Cohen's (1988) rule of thumb and are shown in Table 3.

**Table 3:** Magnitudes of effect size based on the general rule of thumb of Cohen (1988).

**Statistical measure**	**Effect size threshold**		
	**Small**	**Moderate**	**Strong**
Correlation (Pearson's *r*)	0.10	0.30	0.50
ANOVA (*η^2^*)	0.01	0.06	0.14
Cohen's *d*	0.20	0.50	0.80

Australia has a nationwide Australian Curriculum that is implemented and assessed on a State-by-State basis. For example, although the general content of the English curriculum is the same in all states, the details regarding its delivery and assessment are not directly comparable. In addition, the Australian school leaving credentials do not require the study of any mandatory subjects in Years 11 and 12 except for English. Hence, sample sizes for other subjects can readily fall below those needed for reliable generalizations to be made. It is therefore unreasonable to compare students' attitude profiles on a per subject basis. However, courses with similar approaches to learning from both schools can be grouped together into several subject areas—known as Key Learning Areas (KLAs) in Australia—for analysis. This approach becomes more useful when only considering Year 11 and 12 students as the various syllabuses for this stage of school are more consistent between States. For the following analyses we consider five KLAs: English, comprising courses in both English Language and English Literature; Mathematics, comprising both elementary, intermediate and advanced mathematics courses; Sciences, comprising courses in physics, chemistry and biology; Humanities and Social Sciences (HASS), comprising courses such as history, geography, economics, business studies; and Creative and Performing Arts (CAPA), comprising courses such as visual arts, drama, and media arts.

While there are some potentially interesting differences between the attitudinal profiles for the two schools in this study at the individual subject level that require more detailed analysis, we will constrain our results and discussion in this paper to the school-wide composite attitude ratings, the grouping factors of student year group and student gender, and the combined KLA subject attitude ratings. As discussed previously, the crisis-like nature of covid-19 is likely to have a dampening effect on students' long-term attitudes toward their subjects. Hence, we will also restrict our analysis to just six of the attitudinal constructs of the SAS namely difficulty, subject anxiety, self-efficacy, enjoyability, perceived subject relevance, and creativity.

## Results

### School Composite Attitude Ratings

**Figure 2:**
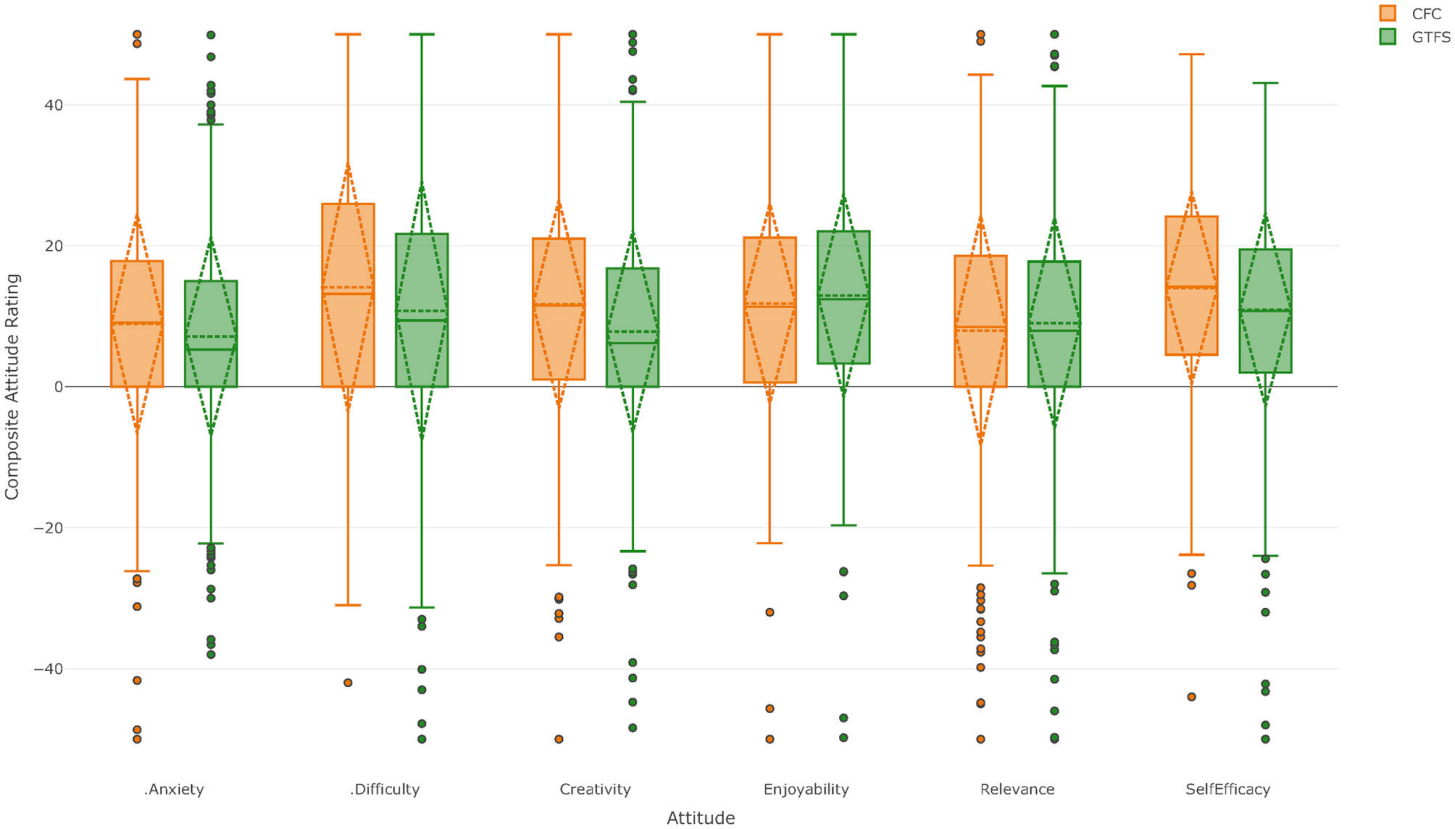
Composite attitude profile for all year 10–12 students at Corroboree Frog College (left hand bars) and Green Tree Frog School (right hand bars).

Figure 2 shows the composite attitude profile of the students at CFC (left hand boxes) and GTFS (right hand boxes) toward all of their academic subjects. For each attitudinal factor, the box represents the interquartile range (IQR), the solid horizontal line indicates the median and the whiskers indicate 1.5 times the IQR. The diamond is centered on the mean and extends one standard deviation in either direction. Circular markers indicate potential outliers in the data. As can be seen, the mean reported attitudes are slightly positive for all attitudinal constructs. This is indicative of a student population who are experiencing the uncertainties of the covid-19 crisis but who nonetheless have a generally positive outlook on school.

The figure suggests that the attitudinal positions held by students at the two schools are in fact very similar, even though the experiences of covid-19 for the students at these schools have been markedly different. This similarity is confirmed by the results of a two-way ANOVA which shows that the effect sizes of any statistically significant differences as a result of attending one school or the other are small. Ratings for self-efficacy by students at CFC were 2.96 points higher [*F*_(1, 820)_ = 9.61, *p* = 0.002, η^2^ = 0.012] than students' ratings at GTFS. Ratings for creativity at CFC were 4.0 points higher [*F*_(1, 809)_ = 16.24, *p* < 0.001, η^2^ = 0.020] than at GTFS. When a student's gender is included in the ANOVA model, a number of apparently statistically significant relationships are revealed; however, none of these results reach the threshold value of eta-squared for a small effect size, as outlined in Table 4, and so they are not considered statistically meaningful.

**Table 4:**
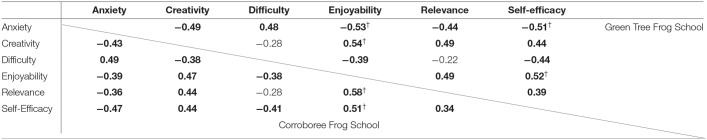
Correlation coefficients between Attitudinal Factors for all students at Corroboree Frog College (below the diagonal) and Green Tree Frog School (above the diagonal).

*Bold text has been used to indicate a moderate correlation (r>0.30). Bold text and a dagger symbol have been used to indicate a strong correlation (r>0.50)*.

A pairwise correlation analysis with a Bonferroni adjustment was performed between each of the attitudinal factors for each of the schools independently. These are shown in Table 4 with the correlations for CFC below the diagonal and those for GTFS above. All correlations were found to be statistically significant at the *p* < 0.001 level. There were no meaningful correlations found between a student's gender or their year group and their CAR for any attitudinal factor for students at either school.

As can be seen in Table 4, creativity correlates favorably with enjoyability (*r*_*CFC*_ = 0.47, *r*_*GTFS*_ = 0.54), relevance (*r*_*CFC*_ = 0.44, *r*_*GTFS*_ = 0.49), and self-efficacy (*r*_*CFC*_ = 0.44, *r*_*GTFS*_ = 0.44) while correlating negatively with perceived difficulty (*r*_*CFC*_ = −0.38, *r*_*GTFS*_ = −0.28), and subject anxiety (*r*_*CFC*_ = −0.43, *r*_*GTFS*_ = −0.49). That is to say, as students' subject creativity ratings increase, their subject anxiety ratings decrease, they find the subject easier and their ratings of self-efficacy, enjoyability and relevance also increase. It can also be seen in the table that enjoyability, relevance and self-efficacy are strongly correlated with each other suggesting that these three attitudes may be related to common influences.

## School Subject Attitude Ratings

### English

**Figure 3:**
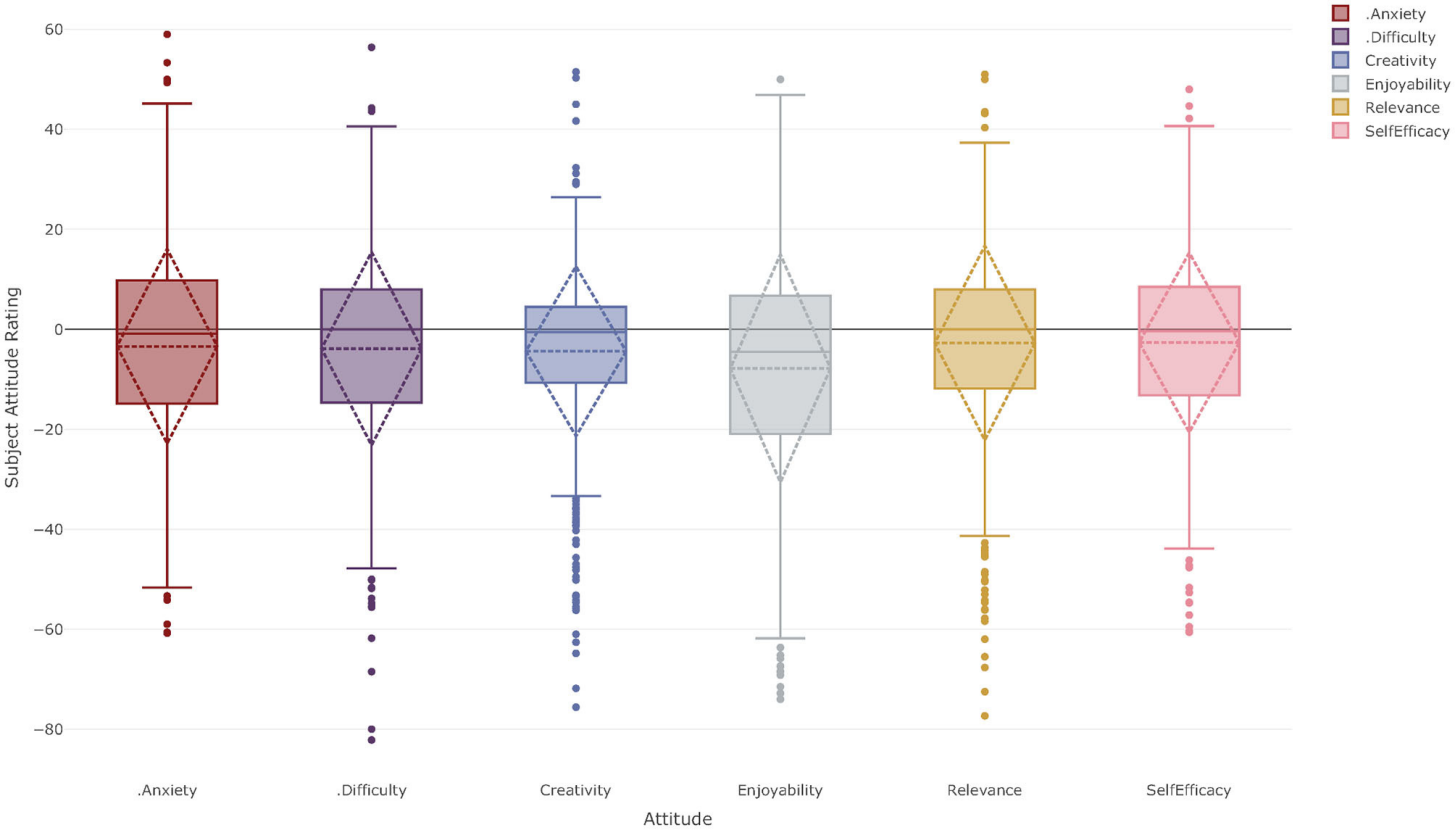
Subject Attitude Profile for year 11–12 students studying at least one course from the English KLA at both Corroboree Frog College and Green Tree Frog School.

Figure 3 shows the subject attitude profile for Year 11 and 12 students for the English KLA. It is important to stress that all students are required to study at least one course from the English KLA as part of their personal curriculum. It is apparent that the students' mean attitude ratings for most of the attitudinal constructs are very similar to the composite attitude ratings for these students as seen in Figure 2, although they are slightly negative. Recall that as the SAR is calculated with reference to a student's CAR a SAR of zero indicates no difference to the CAR; it does not indicate an absolute rating of zero. A two-sided one-sample *t*-test with a 95% confidence interval was carried out to determine if the mean SARs were statistically different from zero, i.e., to determine if the differences between SARs and CARs were statistically significant. Students reported SARs for subject anxiety [*M* = −3.31, *SD* = 19.48, *t*_(578)_ = 4.09, *p* < 0.001, *d* = 0.17], perceived difficulty [*M* = −3.95, *SD* = 19.23, *t*_(579)_ = 4.94, *p* < 0.001, *d* = 0.21], self-efficacy [*M* = −2.69, *SD* = 17.95, *t*_(580)_ = 3.61, *p* < 0.001, *d* = 0.15], relevance [*M* = −2.77, *SD* = 19.55, *t*_(580)_ = 3.42, *p* < 0.001, *d* = 0.14], enjoyability [*M* = −7.96, *SD* = 22.84, *t*_(578)_ = 8.38, *p* < 0.001, *d* = 0.35], and creativity [*M* = −4.41, *SD* = 17.05, *t*_(580)_ = 6.22, *p* < 0.001, *d* = 0.26] that were all <0, statistically significant and of small or borderline small effect size.

Analysis of the inter-attitude correlations for the English KLA shows that the triplet of attitudes identified in the CAR profiles—enjoyability, relevance and self-efficacy—are each strongly correlated with each other for this KLA (*r*_*en*−*re*_ = 0.62, *r*_*en*−*se*_ = 0.66, *r*_*re*−*se*_ = 0.57). In addition, these three attitudes also strongly correlate with students' ratings for creativity (*r*_*cr*−*en*_ = 0.60, *r*_*cr*−*se*_ = 0.57, *r*_*cr*−*re*_ = 0.53). The full correlation matrix can be found in Supplementary Table 2.

### Mathematics

**Figure 4:**
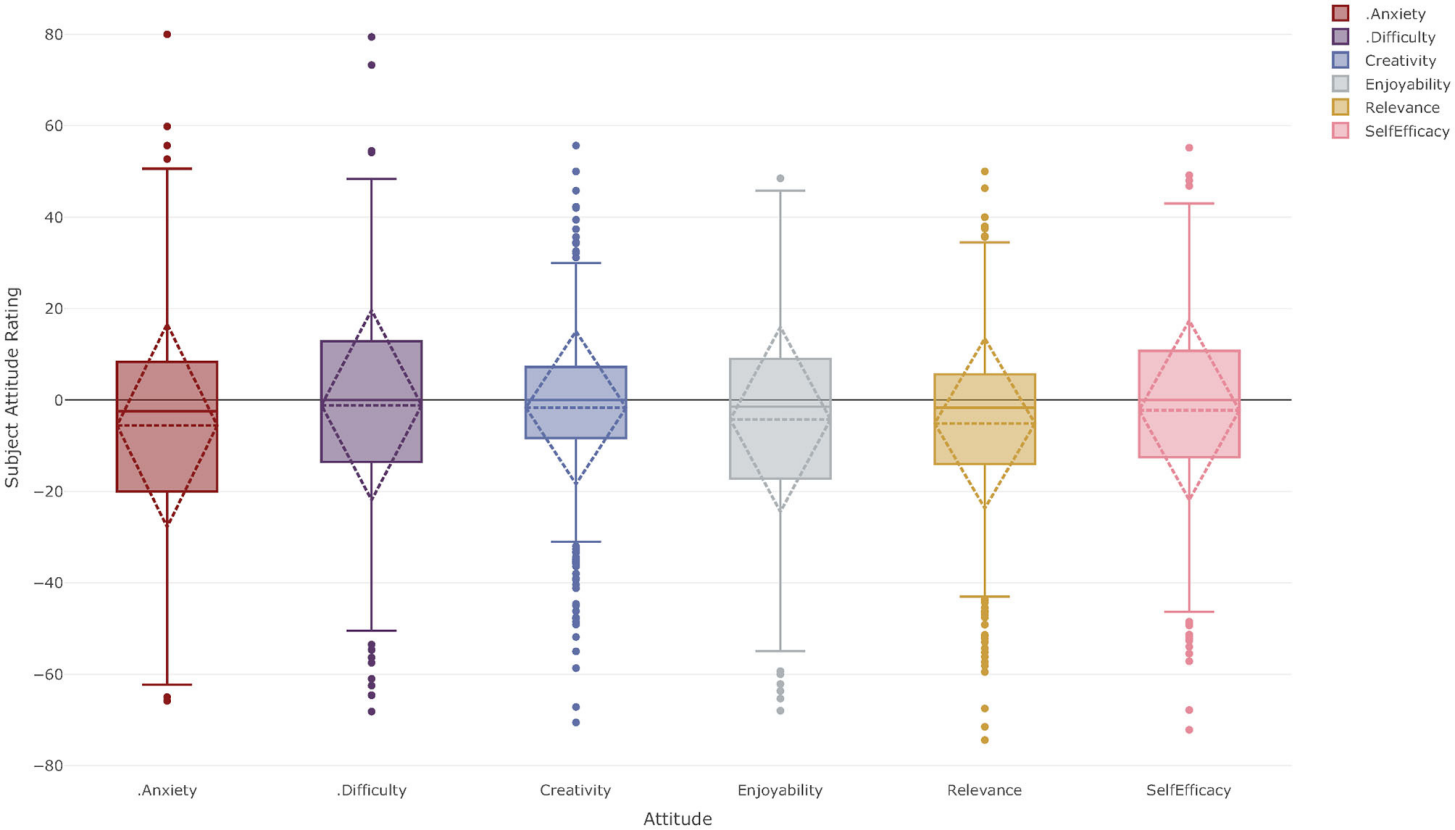
Subject attitude profile for year 11–12 students studying at least one course from the Mathematics KLA at both Corroboree Frog College and Green Tree Frog School.

Figure 4 shows the subject attitude profile for Year 11 and 12 students for courses in the Mathematics KLA. In Australian schools many schools offer mathematics courses at two or three levels of complexity from Year 9 onwards and the study of mathematics is compulsory until the completion of Year 10. In Year 11 and 12, students may elect to study mathematics at one of three levels—elementary level e.g., financial mathematics and algebra; intermediate level e.g., algebra, geometry and non-calculus mathematics; advanced level e.g., calculus mathematics—or not at all. The data presented in Figure 4 therefore represent the SARs of students who have chosen to include some level of mathematics study in their curriculum.

Once again, a two-sided one-sample *t*-test was carried out to determine if the mean SARs for these attitudinal factors were statistically different from the students' CARs. The means of all the SARs were less than these students CARs; however, this difference was not statistically significant for perceived difficulty. Subject anxiety [*M* = −6.26, *SD* = 21.94, *t*_(541)_ = 6.64, *p* < 0.001, *d* = 0.29], creativity [*M* = −2.24, *SD* = 16.01, *t*_(542)_ = 3.26, *p* = 0.001, *d* = 0.14], enjoyability [*M* = −5.16, *SD* = 19.86, *t*_(543)_ = 6.06, *p* < 0.001, *d* = 0.26], relevance [*M* = −5.87, *SD* = 17.98, *t*_(542)_ = 7.60, *p* < 0.001, *d* = 0.33], and self-efficacy [*M* = −2.99, *SD* = 19.40, *t*_(542)_ = 3.59, *p* < 0.001, *d* = 0.15] ratings were all statistically significantly negative compared to their CAR and small effect sizes.

Analysis of the correlations between the attitudinal factors for the Mathematics KLA showed that the correlations between enjoyability, relevance and self-efficacy (*r*_*en*−*re*_ = 0.50, *r*_*en*−*se*_ = 0.58, *r*_*re*−*se*_ = 0.48) are again strong or borderline strong suggesting the interdependence of these three constructs within mathematics. Creativity correlates strongly with enjoyability (*r*_*cr*−*en*_ = 0.49) but only moderately with relevance (*r*_*cr*−*re*_ = 0.36) and self-efficacy (*r*_*cr*−*se*_ = 0.39). The full correlation matrix can be found in Supplementary Table 3.

### Sciences

**Figure 5:**
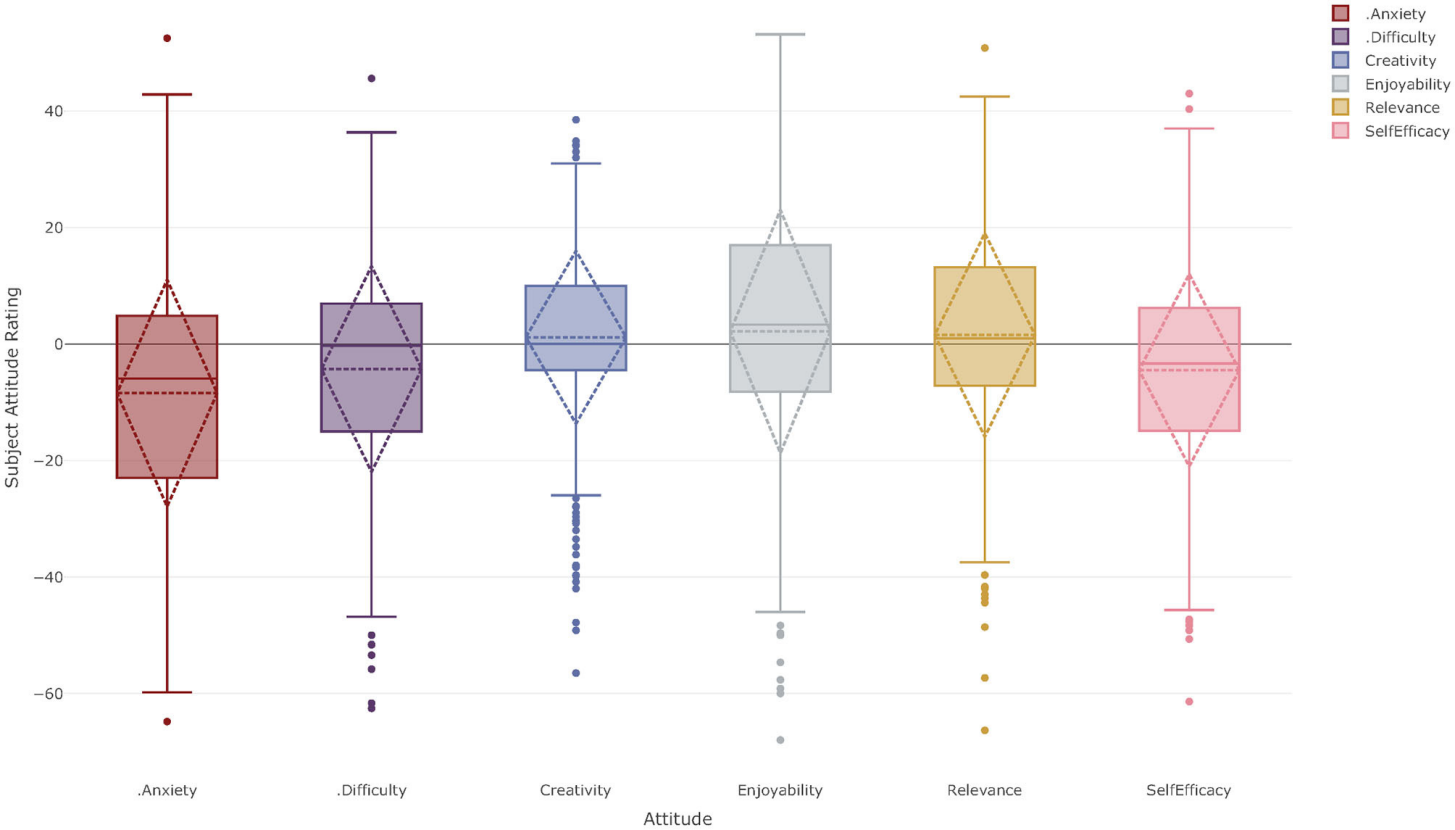
Subject attitude profile for year 11–12 students studying at least one course from the Sciences KLA at both Corroboree Frog College and Green Tree Frog School.

Figure 5 shows the subject attitude profile for Year 11 and 12 students for courses in the Sciences KLA. In Australia, all students study an integrated or general science course until the end of Year 10. In Years 11 and 12, students may choose to study none, one or more courses from the Sciences KLA. These can be domain specific, such as Biology, Chemistry or Physics, or generic, such as Scientific Studies. The data presented in Figure 5 therefore represent the SARs of students that have chosen to include *at least* one science course in their curriculum and the number of data points exceeds the number of students.

As before a two-tailed one-sample *t*-test was performed to determine if the means of the SARs were statistically significantly different to the CARs of the students taking at least one course in the Sciences KLA. SARs for subject anxiety [*M* = −8.86, *SD* = 18.91, *t*_(1, 275)_ = 16.72, *p* < 0.001, *d* = 0.47], perceived difficulty [*M* = −4.60, *SD* = 17.35, *t*_(1, 272)_ = 9.46, *p* < 0.001, *d* = 0.27], and self-efficacy [*M* = −4.85, *SD* = 16.17, *t*_(1, 266)_ = 10.67, *p* < 0.001, *d* = 0.30] were all statistically less than the students' CARs and had small effect sizes. SARs for creativity [*M* = 1.19, *SD* = 14.33, *t*_(1, 270)_ = 2.95, *p* = 0.003, *d* = 0.08], enjoyability [*M* = 2.03, *SD* = 19.98, *t*_(1, 269)_ = 3.62, *p* < 0.001, *d* = 0.10], and relevance [*M* = 1.48, *SD* = 17.14, *t*_(1, 270)_ = 3.09, *p* = 0.002, *d* = 0.09] were all more positive than the CARs for these students and these were all statistically significant but of negligible effect size.

Analysis of the correlations between the different attitudinal factors for the Sciences KLA showed that enjoyability was found to be strongly correlated with both relevance (*r*_*en*−*re*_ = 0.52) and self-efficacy (*r*_*en*−*se*_ = 0.53) which are in turn moderately correlated with each other (*r*_*re*−*se*_ = 0.39). Once again, creativity was borderline strongly correlated with this triplet of attitudinal factors (*r*_*cr*−*en*_ = 0.48, *r*_*cr*−*re*_ = 0.42, *r*_*cr*−*se*_ = 0.48). The full correlation matrix can be found in Supplementary Table 4.

### Humanities and Social Sciences

**Figure 6:**
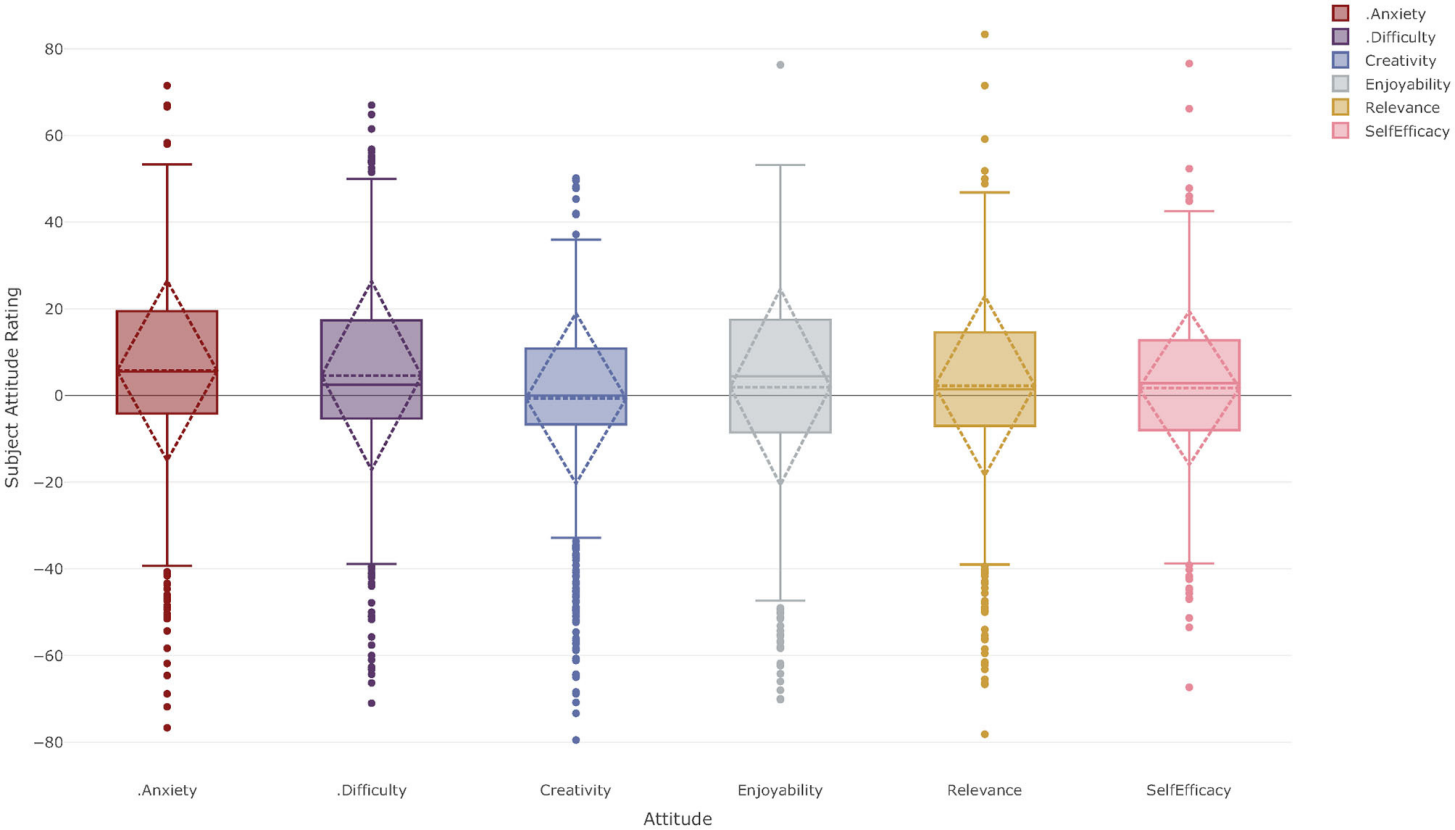
Subject Attitude Profile for Year 11–12 students studying at least one course from the Humanities and Social Sciences KLA at both Corroboree Frog College and Green Tree Frog School.

Figure 6 shows the subject attitude profile for Year 11 and 12 students for courses in the HASS KLA. HASS is arguably the broadest KLA in Australian schools. It includes subjects such as History, Geography, Economics, Business Studies, Legal Studies, Sociology, Psychology and Politics. Students must study at least History and Geography to some degree until Year 10 and in many schools may study one or more elective courses in the HASS KLA from Year 9—possibly including an additional course in History or Geography. Similar to the Sciences, in Years 11 and 12 students may choose to study none, one or more courses from this KLA. The data presented in Figure 6 therefore represents the SARs of students that have chosen to include *at least* one HASS course in their curriculum and the number of data points exceeds the number of students.

As with the other KLAs, a two-tailed one-sample *t*-test was performed to determine if the means of the SARs were statistically significantly different to the CARs of the students taking at least one course in the HASS KLA. The mean SARs were more positive than the students' CAR for all attitudinal factors except creativity. The students' mean creativity rating was very slightly negative [*M* = −0.93, *SD* = 18.76, *t*_(1, 815)_ = 2.10, *p* = 0.04, *d* = 0.05] compared to their CAR but this effect size is negligible. Students reported positive SARs for anxiety [*M* = 6.24, *SD* = 19.09, *t*_(1, 808)_ = 13.91, *p* < 0.001, *d* = 0.33], perceived difficulty [*M* = 5.42, *SD* = 19.90, *t*_(1, 811)_ = 11.60, *p* < 0.001, *d* = 0.27] which were both statistically significant and had a small effect size. SARs for enjoyability [*M* = 1.21, *SD* = 21.23, *t*_(1, 807)_ = 2.41, *p* = 0.02, *d* = 0.06], relevance [*M* = 1.88, *SD* = 19.72, *t*_(1, 815)_ = 4.07, *p* < 0.001, *d* = 0.10], and self-efficacy [*M* = 1.74, *SD* = 16.56, *t*_(1, 808)_ = 4.47, *p* < 0.001, *d* = 0.11] were more positive than their CAR, but had only a negligible effect size.

Analysis of the correlations between the different attitudinal factors for the HASS KLA showed that enjoyability was again found to be strongly correlated with both relevance (*r*_*en*−*re*_ = 0.58) and self-efficacy (*r*_*en*−*se*_ = 0.56) which are also moderately correlated with each other (*r*_*re*−*se*_ = 0.36). Creativity was found to be strongly correlated with enjoyability (*r*_*cr*−*en*_ = 0.54) and moderately correlated with relevance (*r*_*cr*−*re*_ = 0.44) and self-efficacy (*r*_*cr*−*se*_ = 0.40). The full correlation matrix can be found in Supplementary Table 5.

### Creative and Performing Arts

**Figure 7:**
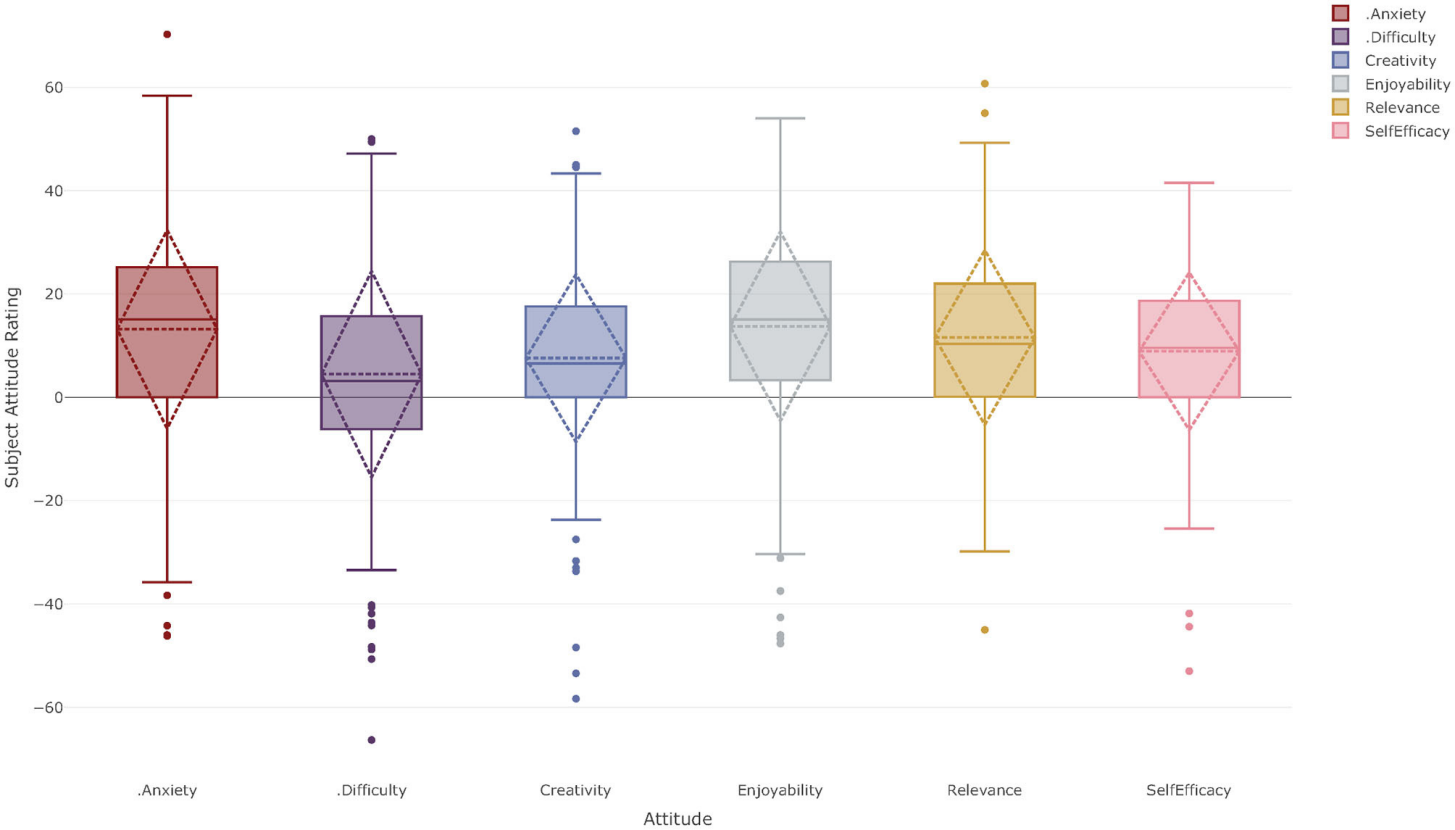
Subject Attitude Profile for Year 11–12 students studying at least one course from the Creative and Performing Arts KLA at both Corroboree Frog College and Green Tree Frog School.

Figure 7 shows the subject attitude profile for Year 11 and 12 students for courses in the CAPA KLA. CAPA is a broad KLA in Australian schools, but often has relatively small enrolments. It includes subjects such as Music, Visual Arts, Drama, and Media Arts. Students must study courses in CAPA until the end of Year 8. From Year 9 onwards, courses in the CAPA KLA tend to be elective only courses. While it is obviously helpful to have studied a CAPA course in Year 9 and 10 if enrolling in a CAPA course in Years 11 and 12, it is not a mandatory requirement. Similar to the HASS KLA and the Sciences, in Years 11 and 12 students may choose to study none, one or more courses from this KLA. The data presented in Figure 7 therefore represent the SARs of students that have chosen to include *at least* one CAPA course in their curriculum and the number of data points exceeds the number of students.

A two-tailed one-sample *t*-test was again performed to determine if the means of the SARs were statistically significantly different to the CARs of the students taking at least one course in the CAPA KLA. The mean SARs were more positive than the students' CAR for all attitudinal factors without exception. Students' SARs for subject anxiety [*M* = 13.57, *SD* = 18.73, *t*_(418)_ = 14.81, *p* < 0.001, *d* = 0.72], creativity [*M* = 7.66, *SD* = 16.38, *t*_(422)_ = 9.61, *p* < 0.001, *d* = 0.47], perceived difficulty [*M* = 4.49, *SD* = 20.38, *t*_(422)_ = 4.53, *p* < 0.001, *d* = 0.22], enjoyability [*M* = 13.99, *SD* = 18.07, *t*_(414)_ = 15.76, *p* < 0.001, *d* = 0.77], relevance [*M* = 11.69, *SD* = 16.77, *t*_(422)_ = 14.31, *p* < 0.001, *d* = 0.70], and self-efficacy [*M* = 8.91, *SD* = 14.72, *t*_(420)_ = 12.40, *p* < 0.001, *d* = 0.61] were more positive than these students' CARs and these were statistically significant with a small to moderate effect size.

The analysis of the correlations between the different attitudinal factors for the CAPA KLA, showed that enjoyability was again found to be strongly correlated with both relevance (*r*_*en*−*re*_ = 0.48) and self-efficacy (*r*_*en*−*se*_ = 0.58), which are also moderately correlated with each other (*r*_*re*−*se*_ = 0.34). Creativity was found to be moderately correlated with enjoyability (*r*_*cr*−*en*_ = 0.47) and self-efficacy (*r*_*cr*−*se*_ = 0.42). The full correlation matrix can be found in Supplementary Table 6.

### Patterns Between KLAs

There appear to be some similarities between the correlation patterns within each of the KLAs discussed above, yet the attitudinal profiles are dissimilar. This is suggestive of the existence of different *types* of students who may choose to preference some KLAs while avoiding others when selecting their courses of study in the final years of school. Examining the correlations between the students' SARs for each of the attitudinal factors between KLAs reveals some statistically significant patterns.

In terms of creativity, students' SARs for the English KLA correlate negatively with the HASS KLA (*r* =-0.34, *p* < 0.001) and the CAPA KLA (*r* = −0.35, *p* < 0.001), while the Sciences KLA has a borderline moderate negative correlation with the HASS KLA (*r* = −0.29, *p* < 0.001). Students' SARs for enjoyability in the Sciences KLA showed borderline moderately negative correlations with the HASS KLA (*r* = −0.28, *p* < 0.001), and the CAPA KLA (*r* = −0.29, *p* = 0.02). The English KLA also showed a moderately negative correlation with the HASS KLA (*r* = −0.28, *p* < 0.001) and the CAPA KLA (*r* = −0.31, *p* < 0.001).

The Mathematics KLA showed SARs with a moderately negative correlation with the HASS KLA (*r* = −0.32, *p* < 0.001) and the CAPA KLA (*r* = −0.29, *p* = 0.01) for self-efficacy while the Sciences KLA showed a borderline strong negative correlation (*r* = −0.48, *p* < 0.001). Considering relevance, there were a number of statistically significant correlations but these all had a small effect size in accordance with Table 3 and so are not reported here.

## Discussion of Results

This exploratory study investigated four key areas. Firstly, students' self-rating of creativity in their independent learning under covid-19 and secondly, how these attitudes vary between schools that have experienced very different impacts of the pandemic. In investigating these first two questions, Figure 2 shows CARs that are very similar for students at both CFC and GTFS. Previous studies (Kennedy, 2018; Kennedy et al., 2018) have shown generally positive attitudes toward both Science and Mathematics and an unpublished longitudinal study following student attitudes to creativity over 3 years (Kaufman et al., 2020) reported that adolescents had generally positive ratings of creative self-efficacy. Therefore, it seems reasonable to suggest that these present data show little impact of covid-19 even though the students at these schools experienced very different social and educational responses to the pandemic. Furthermore, the students' CARs are generally positive, even in a time of societal crisis, which speaks to the resilience and flexibility of the students in adapting to new modalities of pedagogy and independent learning. Phrased differently, the schools' responses to covid-19 appear to have mitigated many of the potentially negative effects on student attitudes and students continue to hold generally positive attitudes toward the academic aspects of school as a whole.

As previously noted, students' attitudes toward creativity and self-efficacy were slightly more positive at CFC than at GTFS. While it might seem reasonable to suggest that the continuing uncertainty surrounding covid-19, reduced direct and immediate access to teaching staff, and the extended period spent engaged in online learning by students at GTFS could be a contributing factor to these differences, it is impossible to say for certain whether or not this is the case. Further research is required in this area to determine the nature of the attitude profiles at the two schools once they return to a semblance of normality.

Our third research question looked at other attitudinal constructs with the purpose of identifying areas of commonality across key learning areas of the curriculum. Kennedy et al. (2018) showed that the attitudinal factors of enjoyability, self-efficacy and relevance were closely linked for Year 7 students studying Science and Mathematics. As we have shown above (and also in Supplementary Tables 2–6), this triplet of attitudes continues to be moderately to strongly correlated with each other across all five KLAs for the Year 11 and 12 students' SARs analyzed in this study. These patterns are interesting as they suggest that there may be feedback mechanisms present between these various attitudinal factors that are similar to each other across KLAs or skill domains. Furthermore, that this same triplet of attitudes is seen in both early and late secondary students suggests significant potential for future action and offer opportunities for further research into various academic interventions that could help to shape students' attitudes and assist in developing a more positive view of self-concept for students.

However, while there are common patterns within the KLAs, Figures 3–7 also show differences in the nature of the SARs between KLAs. Figure 4 suggests students feel slightly more anxious about their English course and slightly less confident about it than they do about the other courses they study in general. They also identified English as being slightly more difficult than their courses in general and that they were able to be slightly less creative in English. Interestingly they report that English is very slightly less relevant than their courses as a whole and much less enjoyable. This may be a consequence of the mandatory nature of the English KLA or it may be a result of the curriculum design or a combination of factors. This is an area that warrants further research. The Mathematics KLA shows a somewhat similar pattern to English; students are slightly more anxious about their Mathematics course and find it less interesting and relevant than the mean of the remainder of their courses. They also find it slightly more difficult than their other courses and report slightly lower creativity and self-efficacy toward it. Further research is clearly needed in this area.

Similarly, the Science KLA, HASS KLA, and CAPA KLA show different profiles to each other and that are different to Mathematics and English. The pairwise correlation analyses performed between each of the KLAs for the different attitudinal factors, included only those students studying at least one course from the two KLAs of interest. While there are, for example, many students studying courses in HASS and in the Sciences, the statistically meaningful correlations—those that are both statistically significant and of moderate effect size or greater—for a given attitudinal factor tend to be negative correlations. It appears that students hold different attitudes toward the different domains of their curriculum. This suggests that a one size fits all approach to learning does not exist across the different KLAs and that any potential interventions may need to be KLA specific or focused.

**Figure 8:**
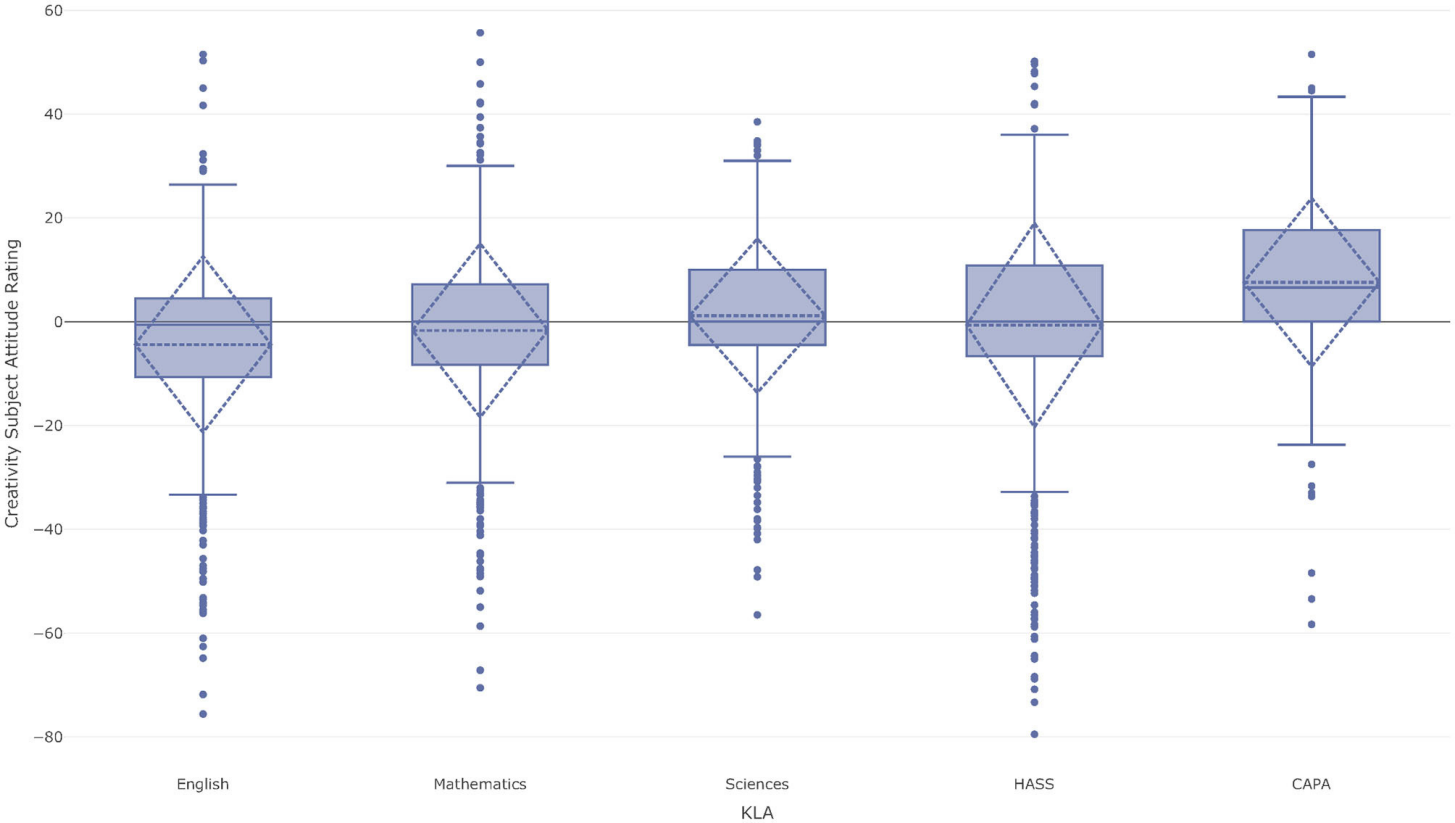
Attitude profile for creativity for Year 11–12 students studying at least one course from the English, Mathematics, Sciences, HASS, or CAPA KLAs at both Corroboree Frog College and Green Tree Frog School.

Our final research question aimed to investigate the domain specificities of students' perception of creativity between key learning areas. One big debate is whether creativity is domain-specific or domain general. In this study we added the additional construct of creativity to the SAS and in doing so, we carefully worded the item (see Supplementary Table 1) to describe the *action* of learning creatively. In Figures 3–7 we see that students' ratings of creativity compared to their CAR are different across the KLAs. These creativity SARs are shown again in Figure 8 side by side. As English is a mandatory subject it can be used as a comparative baseline for a pairwise correlation analysis between the SARs for the KLAs. This shows a moderate negative correlation between English and CAPA (*r* = −0.35, *p* < 0.001) and English and HASS (*r* = −0.34, *p* < 0.001). There is also a small negative correlation between English and Mathematics (*r* = −0.21, *p* < 0.001) and English and Science (*r* = −0.19, *p* = 0.02). These various correlations together with Figure 8 suggest that students have different conceptions of and opportunities for creativity in the different domains of their curriculum and that therefore approaches to teaching creativity need to be KLA specific.

Finally, we have also found that creativity is closely correlated with the triplet of attitudes previously discussed across all of the KLAs analyzed. This is particularly interesting because, while the triplet of attitudes measures value based or perceived attitudinal constructs, our creativity construct is action based. That is, creativity, as defined and described in this paper, is a teachable skill and a measurable approach to learning. Therefore, creativity may have some potential to be a mechanism to affect the positive changes in students' attitudes discussed earlier and may in turn be able to assist in reducing subject anxiety and students' perceptions of relative difficulty.

## Conclusions and Further Work

Overall, we have found that even in a time of great uncertainty and societal upheaval, students at these schools reported generally positive attitudes toward their schooling as demonstrated by the students' CARs. This is pattern is consistent with the findings of Kennedy et al. (2018) who investigated the attitudes of Year 7 students toward Science and Mathematics in New South Wales. Furthermore, we found that there were very few statistically significant differences between the composite attitude profiles of both schools even though the two schools have had vastly different experiences of covid-19 and have been impacted in very different ways.

Even though this study presents the results of a small sample of students from just two schools, examining the students' subject attitude profiles on a KLA basis has shown that there are some significant differences between students' attitudes toward the different KLAs. This is likely indicative of the different approaches to learning present in these areas as well as students expressing a personal preference for one particular learning approach over another. While there are undoubtedly come embedded cultural differences between the two groups of students which may have had some influence on students' responses, it is clear that a one size fits all approach to learning design is not appropriate. Therefore, there are opportunities for further research here to investigate how the differing approaches to learning present in the classroom or online in one KLA can inform and shape learning across the curriculum as a whole.

We have also shown that within each KLA there is a particularly well-correlated triplet of attitudes—namely self-efficacy, relevance, and enjoyability—that is a consistent group across KLAs. This value and perception-based group correlates well in each case with students' ratings for opportunities for creative learning, consistent with a wide body of work on the relationship of views about the self and creativity (Karwowski and Kaufman, 2017). Thus, we believe that the skills and processes inherent in creativity may, when applied in a KLA relevant context, offer a window into effecting positive change in students' attitudes across the curriculum.

These findings, although exploratory, give an insight into the minds of adolescents at an unprecedented time in their lives. Although the disruptions students have faced this year in regard to the covid-19 pandemic are likely wider ranging than we have been able to capture with the SAS instrument, our finding of positive student attitudes is reassuring. In terms of creativity, students were learning new processes in a new environment, requiring for many a new set of learning skills that in turn required open, positive and flexible attitudes. Adjusting to any one of these can be challenging, never mind all at once. Despite the new external constraints on their educational experience, students at these schools reported adaptive rather than maladaptive attitudes and behaviors. A willingness to explore new and useful ways of learning in such times is very promising. Creativity has already shown links to a number of socioemotional traits and abilities that are particularly important during COVID times, such as enabling growth after trauma (Forgeard, 2013) and reducing stress (Byron et al., 2010). Indeed, there have been many reports of people specifically turning to creativity to cope with the many challenges of the pandemic (Kapoor and Kaufman, 2020). There is also now solid evidence that helping students facilitate their learning in difficult and new circumstances can be added to this list of ways that creative can help out during these challenging times.

## Data Availability Statement

The datasets presented in this article are not readily available because their use is subject to approval by the relevant ethics committee. Requests to access the datasets should be directed to john.kennedy@unisa.edu.au.

## Ethics Statement

The studies involving human participants were reviewed and approved by The University of South Australia Human Research Ethics Committee. Written informed consent to participate in this study was provided by the participants' legal guardian/next of kin.

## Author Contributions

JKe, TP, and WJ supervised the collection of data. JKe analyzed the data and interpreted it with HK. The initial draft was written by JKe, TP, and SL with additional contributions by DC and JKa. All authors contributed to this article and approved the submitted version.

## Conflict of Interest

The authors declare that the research was conducted in the absence of any commercial or financial relationships that could be construed as a potential conflict of interest.
