# Supplementary material for: Secondary Education in COVID Lockdown: More Anxious and Less Creative—Maybe Not?
Source: Front Psychol. 2021 Feb 22;12:613055. doi: 10.3389/fpsyg.2021.613055 (PMC7937704; doi:10.3389/fpsyg.2021.613055)
Supplement: Supplementary file 1 [file Table_1.DOCX]

Supplementary Material

# Supplementary Tables

Supplementary Table 1: Item wording for each of the Attitudinal Factors in the School Attitudes Survey. The placeholders <SUBJECT> and <OCCUPATION> are replaced at run-time with the participant’s actual subject names or an appropriate occupation that that subject might reasonably lead towards.

| Attitudinal Factor | Item Wording | Left Hand Indicator | Right Hand Indicator |
| --- | --- | --- | --- |
| Subject Anxiety* | When I study <SUBJECT> I feel: | Worried | Relaxed |
| Creativity | When I study <SUBJECT> I am able to develop new and useful ways of independent learning | Strongly Disagree | Strongly Agree |
| Difficulty* | I struggle with completing the assignments for <SUBJECT> class. | Strongly Disagree | Strongly Agree |
| Enjoyability | I think <SUBJECT> is: | Boring | Enjoyable |
| Self-Efficacy | I think I am very good at <SUBJECT> | Strongly Disagree | Strongly Agree |
| Career Usefulness | A job as a <OCCUPATION> would be interesting. | Strongly Disagree | Strongly Agree |
| Personal Usefulness | For my planned career, knowledge of school <SUBJECT> will be: | Worthless | Priceless |
| Relevance | <SUBJECT> helps to make life better. | Strongly Disagree | Strongly Agree |
| Intentions^†^ | I am very likely to enroll on a <SUBJECT> course in Year 11.  I am very likely to enroll on a <SUBJECT> course after school | Strongly Disagree | Strongly Agree |

* These items are reverse keyed in the analyses. ^†^Only one of these item wordings is displayed to the student depending on whether they are a Year 10 student (upper wording) or a Year 11 or 12 student (lower wording)

Supplementary Table 2: Correlation coefficients between Attitudinal Factors for all Year 11 and 12 students at both schools studying a course from the English KLA. Bold text has been used to indicate a moderate correlation (*r*>0.30). Bold text and a dagger symbol have been used to indicate a strong correlation (*r*>0.50).

|  | Anxiety | Creativity | Difficulty | Enjoyability | Relevance | Self-Efficacy |
| --- | --- | --- | --- | --- | --- | --- |
| Anxiety |  |  |  |  |  |  |
| Creativity | -0.50^†^ |  |  |  |  |  |
| Difficulty | 0.57^†^ | -0.46 |  |  |  |  |
| Enjoyability | -0.57^†^ | 0.60^†^ | -0.56^†^ |  |  |  |
| Relevance | -0.45 | 0.53^†^ | -0.45 | 0.62^†^ |  |  |
| Self-Efficacy | -0.62^†^ | 0.57^†^ | -0.60^†^ | 0.66^†^ | 0.57^†^ |  |

Supplementary Table 3: Correlation coefficients between Attitudinal Factors for all Year 11 and 12 students at both schools studying a course from the Mathematics KLA. Bold text has been used to indicate a moderate correlation (*r*>0.30). Bold text and a dagger symbol have been used to indicate a strong correlation (*r*>0.50).

|  | Anxiety | Creativity | Difficulty | Enjoyability | Relevance | Self-Efficacy |
| --- | --- | --- | --- | --- | --- | --- |
| Anxiety |  |  |  |  |  |  |
| Creativity | -0.42 |  |  |  |  |  |
| Difficulty | 0.68^†^ | -0.40 |  |  |  |  |
| Enjoyability | -0.56^†^ | 0.49 | -0.47 |  |  |  |
| Relevance | -0.46 | 0.36 | -0.41 | 0.50^†^ |  |  |
| Self-Efficacy | -0.76^†^ | 0.39 | -0.65^†^ | 0.58^†^ | 0.48 |  |

Supplementary Table 4: Correlation coefficients between Attitudinal Factors for all Year 11 and 12 students at both schools studying a course from the Sciences KLA. Bold text has been used to indicate a moderate correlation (*r*>0.30). Bold text and a dagger symbol have been used to indicate a strong correlation (*r*>0.50).

|  | Anxiety | Creativity | Difficulty | Enjoyability | Relevance | Self-Efficacy |
| --- | --- | --- | --- | --- | --- | --- |
| Anxiety |  |  |  |  |  |  |
| Creativity | -0.39 |  |  |  |  |  |
| Difficulty | 0.54^†^ | -0.35 |  |  |  |  |
| Enjoyability | -0.38 | 0.48 | -0.34 |  |  |  |
| Relevance | -0.31 | 0.42 | -0.33 | 0.52^†^ |  |  |
| Self-Efficacy | -0.64^†^ | 0.48 | -0.53^†^ | 0.53^†^ | 0.39 |  |

Supplementary Table 5: Correlation coefficients between Attitudinal Factors for all Year 11 and 12 students at both schools studying a course from the HASS KLA. Bold text has been used to indicate a moderate correlation (*r*>0.30). Bold text and a dagger symbol have been used to indicate a strong correlation (*r*>0.50).

|  | Anxiety | Creativity | Difficulty | Enjoyability | Relevance | Self-Efficacy |
| --- | --- | --- | --- | --- | --- | --- |
| Anxiety |  |  |  |  |  |  |
| Creativity | -0.27 |  |  |  |  |  |
| Difficulty | 0.55^†^ | -0.11 |  |  |  |  |
| Enjoyability | -0.42 | 0.54^†^ | -0.26 |  |  |  |
| Relevance | -0.29 | 0.44 | -0.15 | 0.58^†^ |  |  |
| Self-Efficacy | -0.57^†^ | 0.40 | -0.46 | 0.56^†^ | 0.36 |  |

Supplementary Table 6: Correlation coefficients between Attitudinal Factors for all Year 11 and 12 students at both schools studying a course from the CAPA KLA. Bold text has been used to indicate a moderate correlation (*r*>0.30). Bold text and a dagger symbol have been used to indicate a strong correlation (*r*>0.50).

|  | Anxiety | Creativity | Difficulty | Enjoyability | Relevance | Self-Efficacy |
| --- | --- | --- | --- | --- | --- | --- |
| Anxiety |  |  |  |  |  |  |
| Creativity | -0.24 |  |  |  |  |  |
| Difficulty | 0.57^†^ | -0.36 |  |  |  |  |
| Enjoyability | -0.42 | 0.47 | -0.41 |  |  |  |
| Relevance | -0.38 | 0.23 | -0.33 | 0.48 |  |  |
| Self-Efficacy | -0.46 | 0.42 | -0.43 | 0.58^†^ | 0.34 |  |
